# A Comprehensive Study on the Physicochemical Characterisation of Plant-Based By-Products

**DOI:** 10.3390/ma18092054

**Published:** 2025-04-30

**Authors:** Filipa Costa, Daniel Mendanha, Joana M. Gomes, Juliana A. S. A. Oliveira, Cecília Ribeiro, Ana Francisca Miranda, José R. M. Barbosa, Olívia Salomé G. P. Soares, Manuel Fernando R. Pereira, Jorge Santos, Beatriz Freitas, Carla J. Silva

**Affiliations:** 1 CITEVE—Portuguese Technological Center for the Textile and Clothing Industry, Rua Fernando Mesquita 2785, 4760-034 Vila Nova de Famalicão, Portugal; afcosta@citeve.pt (F.C.);; 2 CeNTI—Centre for Nanotechnology and Advanced Materials, Rua Fernando Mesquita 2785, 4760-034 Vila Nova de Famalicão, Portugal; 3 LSRE-LCM—Laboratory of Separation and Reaction Engineering-Laboratory of Catalysis and Materials, Faculty of Engineering, University of Porto, Rua Dr. Roberto Frias, 4200-465 Porto, Portugalosgps@fe.up.pt (O.S.G.P.S.);; 4 ALiCE—Associate Laboratory in Chemical Engineering, Faculty of Engineering, University of Porto, Rua Dr. Roberto Frias, 4200-465 Porto, Portugal; 5 LEPABE—Faculty of Engineering, University of Porto, Rua Dr. Roberto Frias, 4200-465 Porto, Portugal; 6 ARCP—Rede de Competência em Polímeros, Rua Dr. Júlio de Matos 828 882, 4200-355 Porto, Portugal

**Keywords:** circular economy, waste management, by-products, physicochemical properties, antioxidant activity, sustainability

## Abstract

The rapid growth of the global population has led to significant environmental impacts, driven by the unsustainable extraction of resources and waste generation. To address these challenges, the valorisation of by-products from different industries is crucial for maximising resource efficiency, reducing waste, and promoting sustainable practices. In this study, a comprehensive characterisation of the physicochemical properties of plant-based by-products, including rice husk (RH), oregano stalks (OS), eucalyptus leaves (EL), and almond shells (AS), was conducted. The analyses of the residues showed that, despite the similarities regarding cellulose and lignin content in all materials, RH and OS present a higher cellulose content, while EL and AS contain a greater percentage of oils. Additionally, calcium and potassium were identified as the metals at higher concentrations in all residues. The EL and RH present significant hydrophobic properties compared to the other analysed residues, showcased by their lower wettability. The morphological analyses of the waste residues revealed that OS and RH particles exhibit fibrous characteristics with heterogeneous sizes, while EL is a blend of fibrous and amorphous particles, and AS is composed of smaller particles with irregular shapes. All the residues retained their antioxidant properties over a 12-month storage period, with no degradation due to grinding. The composition and physicochemical properties of these residues highlight their potential to be used in distinct industries, including construction, transport, and textiles, promoting a circular economy and supporting a more sustainable environment.

## 1. Introduction

The continuous growth of the global population has placed enormous pressure on the environment through the unsustainable extraction of resources and associated waste generation [1]. The depletion of resources, greenhouse gas emissions, and global climate change have brought to the forefront the necessity and urgency of changing our consumption patterns, production processes, and overall approach to resource management. Currently, waste management is a global challenge, considering the substantial resources and energy required for its treatment or disposal, which highlights the need for innovative and more efficient management strategies [2]. The concept of a circular economy has emerged as an alternative to the “extraction–production–disposal” paradigm of the linear economy, currently applied on a large scale in the industrial sectors [3]. The circular model aims to minimise waste and make the most of resources by extending product life cycles and promoting reuse, such as the incorporation of industry waste in different sectors [3,4]. This approach aims to reduce waste and by-product generation by transforming them into value-added products, minimising the environmental impact while supporting economic development [5].

The rapid expansion and development of the agricultural and forestry sectors have led to a substantial increase in waste produced [6]. According to estimates, around 20.3 billion tons of agricultural waste were generated globally in 2019, with projections to increase in the coming years [7,8]. Typically, these wastes and residues have been considered low-value by-products, being in some cases repurposed as livestock feed or fuel for domestic heating. Nevertheless, there is growing interest in finding more sustainable and innovative uses for these waste streams, aiming to give them a second life [9]. This shift not only helps to reduce their environmental impact but also unlocks new economic opportunities by transforming waste into valuable resources for various industries. Plant-derived waste and by-products, such as peels, leaves, seeds, and pomace, are generated during food/materials production and processing. Research shows they can be valorised by extracting valuable nutrients like proteins, vitamins, fatty acids, and phenolic compounds [10]. Of the various plant-based resources currently explored, eucalyptus stands out as one of the most widely used species in the paper-making industry. However, eucalyptus is a controversial plant due to high water consumption, nutrient depletion, and its allopathic effects [11]. Furthermore, while eucalyptus leaves (EL) have recently gained attention for the extraction of their oil, which has been used in the preparation of chewing gums, candies, and antimicrobial packaging, they are still largely considered a low-value by-product and are often discarded [12]. Leaf biomass can constitute up to 20–30% of the total eucalyptus biomass, amounting to one to three tons per hectare annually, depending on plantation density and management practices.

In parallel, agricultural wastes, such as rice husk (RH) and almond shells (AS), are significant contributors to the global by-product landscape. RH is one of the primary agricultural wastes produced in rice-cultivating countries, accounting for approximately 20% of rice weight [13]. The annual global rice production exceeds 750 million tons of grain, generating around 150 million tons of husk. The composition of RH depends directly on the rice type, soil conditions, and climate, and its pyrolysis can be used to produce derivatives, such as biochar, activated carbon, and silica, that can be used in various industries, from construction to the personal care industry [13,14]. Similarly, AS are currently largely discarded or incinerated. Considering that global almond production was estimated at around 3.5 million metric tons in 2020, it is estimated that approximately 1.75 million metric tons of AS are produced annually [15]. Recently, AS has been explored as a filler in biocomposites as an alternative to fossil-based materials, but their composition and potential properties are still unclear [16]. Additionally, studies have investigated the use of AS in plastic materials, demonstrating improvements in mechanical properties and highlighting their potential as a low-cost, eco-friendly reinforcing agent [17].

Nowadays, medicinal and aromatic plants are increasingly being cultivated and processed for their medicinal, cosmetic, and herbal properties. However, only certain parts of the plants, such as the fruit, root, leaf, and flower, are valued for obtaining natural bioactive compounds. The processing of these materials yields a substantial number of by-products, such as hydrolates and solid residues generated during the essential oil extraction process, as well as post-harvest by-products such as branches, stalks, and stems that are not commercially viable. These aromatic residues comprise an estimated 20 million tons worldwide each year [18]. Thus, these by-products accumulate as waste and are often left on the fields, polluting the environment, and they are often incinerated, contributing to additional CO_2_ emissions. Oregano (*Origanum* spp.) is one of the world’s most recognised aromatic plants due to its use in culinary and essential oils. The processing of over 10 tons of oregano showed that only 46% of the produced product was commercially acceptable, while 54% was considered a by-product [19]. Therefore, it is imperative to study the potential of these by-products and identify their physicochemical profiles to generate newly value-added materials [11].

For these reasons, it is urgent that new strategies be developed for the reconversion of industries’ plant-based sub-products or improvement of the current ones, such as microbial conversion and pectin and phenolic extractions. The by-products (EL, RH, AS, and oregano stalks) were chosen for their abundance in Portuguese industry and their previously identified characteristics. However, a limited understanding of their physicochemical properties remains the main challenge for their further application and exploitation, limiting their application and valorisation. In this study, we conduct a comprehensive characterisation of the chemical composition and key physicochemical properties of selected industry by-products. Specifically, we analyse elemental composition, wettability, and antioxidant characteristics to assess their functional properties. These analyses intend to showcase potential applications for the different materials, opening new application pathways. This research provides important insights into the valorisation of plant-based by-products, supporting the transition towards a more sustainable and circular economy.

## 2. Materials and Methods

### 2.1. Materials and Chemicals

OS stalks were generously supplied by Ervital Lda. (Viseu, Portugal), EL by CAIMA S.A. (Santarém, Portugal), RH by Novarroz S.A. (Aveiro, Portugal), and AS was provided by Quinta da Fonte do Lobo Lda. (Porto, Portugal). The 2,2′-azino-bis(3-ethylbenzothiazoline-6-sulfonic acid) diammonium salt (ABTS, CAS 30931-67-0) was purchased from Sigma-Aldrich (Saint Louis, MO, USA).

### 2.2. Pre-Treatment and Storage of Residues

The plant-based residues were stored with low or absent luminosity, at room temperature, and with a relative humidity below 60% immediately after being received. EL underwent a drying process using a stationary conveyor-belt dryer (Roqtunnel Infradryer T3080E, SROQUE, Oliveira São Mateus, Portugal) at 60 °C for 3 h. After drying, all the residues were stored under the same conditions as the other samples.

**Figure 1:**
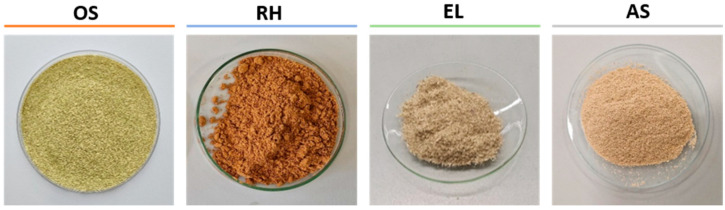
Plant-based by-products—oregano stalks (OS), rice husk (RH), eucalyptus leaves (EL), and almond shells (AS)—at their final granulometry of 0.25 mm.

To standardise particle size, all the residues were ground using a pilot-scale cutting mill (SM 300, Retsch, Haan, Germany) to achieve a final granulometry of 0.25 mm (Figure 1). To facilitate this process, the plant-based by-products were sequentially sized down to 10 mm, 4 mm, 1 mm, and 0.25 mm.

To evaluate the influence of different storage conditions on the residues’ physicochemical properties, samples were stored after a drying process, either without grinding or ground to a final granulometry of 0.25 mm. The by-products were kept in low-light or dark conditions and at room temperature, as described above.

### 2.3. Physicochemical and Morphological Characterisation

#### 2.3.1. Fourier Transform Infrared Spectroscopy (FTIR)

For the chemical characterisation of the samples, FTIR was conducted; the spectra were collected using a VERTEX 70 FTIR spectrophotometer (Bruker, Billerica, MA, USA) equipped with an ATR (attenuated total reflectance) cell with a diamond crystal (A225/Q PLATINUM ATR). The spectra were acquired in the range from 500 to 4000 cm^−1^ with a resolution of 4 cm^−1^.

#### 2.3.2. Thermogravimetric and Differential Scanning Calorimetry Analysis (TGA-DSC)

The thermal stability and thermal behaviour were evaluated through thermogravimetric and differential scanning calorimetry in a one-step analysis using a TGA-DSC, Netzsch STA 409 PC Luxx^®^ apparatus (Netzsch-Gerätebau, Selb, Germany). The TGA-DSC analyses were performed using alumina crucibles with approximately 10 mg of plant-based by-products that were analysed in a temperature range of 50–900 °C with a heating rate of 10 °C min^−1^. The analyses were carried out using an inert atmosphere (N_2_ gas) during the heating step, in an isothermal step of 7 min at 900 °C, and in an oxidative atmosphere (air) at 900 °C for 13 min.

#### 2.3.3. Elemental Analysis (EA)

Elemental analysis of carbon (C), hydrogen (H), nitrogen (N), sulfur (S), and oxygen (O) was performed mostly following a methodology largely aligned with the ISO 16948:2015 standard for solid biofuels, except for the drying step and the determination of ash content [20]. The plant-based by-product samples were first dried at 60 °C for 3 h, as mentioned previously. The effectiveness of this drying method was validated by different techniques presented in this study, which allowed us to determine whether the elemental analysis (EA) was performed on a dry-free basis (without moisture and with ash) or an as-received basis (including moisture). After drying, triplicate sample portions were prepared by weighing between 1.5 and 2.0 mg of each material. The samples intended for C, H, N, and S determination were encapsulated in tin wrappers, while those for oxygen determination were encapsulated in silver wrappers. Elemental analysis was conducted using a Vario Micro CHNS analyser operating at 1050 °C for C, H, N, and S measurements, and an OXY cube analyser operating at 1450 °C for O measurements. Calibration of the equipment was performed using the following certified reference materials: sulphanilamide (Elementar, DE) for C, H, N, and S, and benzoic acid (Sigma-Aldrich, Saint Louis, MO, USA) for O. Then, the elemental composition of each sample was determined through the analysis software coupled with the instruments, which measured peak areas corresponding to each element. Statistical treatment was applied to obtain the mean values from the triplicate measurements for each element and material, with results expressed in weight percentage (wt.%).

#### 2.3.4. Chlorine Content Determination by Ionic Chromatography (IC)

The presence of chlorine in biomass is an important parameter to be monitored since it could be responsible for corrosion, slagging, and fouling in combustion systems [21,22]. Typically, this element appears as a microelement in biomass, but even at a low concentration, it can contribute to the phenomena mentioned before. Therefore, the chlorine content in the plant-based materials characterised in this study was determined using IC, which was conducted following a similar analysis methodology reported elsewhere [23]. Firstly, the plant-based materials underwent a digestion process using a Milestone START D microwave digester, wherein 25 mg of each material was mixed with 10 mL of concentrated HNO3 and then placed in the microwave digester, which carried out the digestion process at 180 °C and 1000 W for 30 min. Then, the digested samples were slightly diluted, and the pH was adjusted to prevent damage to the IC apparatus, which consisted of a Metrohm 881 Compact IC Pro apparatus (Herisau, Switzerland) equipped with a Metrosep A Supp 7 anionic exchange column (250 mm × 4.0 mm). Ultra-pure water was used as solvent to prepare a solution of 3.2 mM Na_2_CO_3_ from Panreac and 1 mM NaHCO_3_ from Panreac to be used as mobile phase (0.7 mL/min). The calibration curve was prepared using NaCl from José Manuel Gomes dos Santos, LDA. The limit of detection and quantification of anion chlorine was 0.452 and 1.36 ppm, respectively.

#### 2.3.5. Inductively Coupled Plasma-Optical Emission Spectroscopy (ICP-OES)

Considering that some of the by-products could contain inorganic matter, ICP-OES analyses were performed to identify and quantify metal elements. The materials were digested following the same method used for chlorine content determination by IC analyses.

The obtained liquid samples were diluted for ICP-OES analysis. The detection method was optical emission spectroscopy, and the instrument used was an ICP–OES ICAP 7400 THERMO with a nebulising system. Two periodic table mixes for ICP (mix 1 and 2) from Sigma-Aldrich (Saint Louis, MO, USA) were used to prepare standards that were used to obtain calibration curves. Mix 1 contains the following list of elements: Ag, Al, As, B, Ba, Be, Bi, Ca, Cd, Co, Cr, Cs, Cu, Fe, Ga, In, K, Li, Mg, Mn, Na, Ni, P, Pb, Rb, S, Se, Si, Sr, Te, Tl, V, and Zn; mix 2 contains Au, Ge, Hf, Ir, Mo, Nb, Pd, Pt, Re, Rh, Ru, Sb, Sn, and Ta.

#### 2.3.6. Wettability

The wettability of the samples was evaluated by measuring the time needed for the sample to fully penetrate the surface of distilled water. For each test, 5 g of the sample was gently placed on top of 100 mL of distilled water at 20 °C, ensuring minimal disturbance of the water surface. The time taken for complete penetration of the powder into the water was recorded. Three replicates were performed for each sample.

#### 2.3.7. Contact Angle

Contact angle measurements were carried out using the sessile drop method with a goniometer (OCA 15, DataPhysics, Filderstadt, Germany) and the SCA20 software (version 5.0.32, DataPhysics, Filderstadt, Germany). Ultra-pure water was used in the tests. The by-products’ powders were pressed into circular pellets with the assistance of an Atlas 15T manual pellet press (Specac^®^, Orpington, UK). During the test, a syringe dispensed a drop of water onto the pellet’s surface with a specified volume of 0.5 μL. Subsequently, the camera captured the image of the drop, and with the help of the software application, the contact angle was determined from the tangent to the drop’s profile. Three replicates were performed for each sample.

#### 2.3.8. Scanning Electron Microscopy (SEM)

The SEM images for the analysis of the powder morphology of the samples were acquired using an FEI Quanta 400FEG ESEM/EDAX Genesis X4M (Thermo Fisher Scientific, Hillsboro, OR, USA). The samples were pre-coated with a gold/palladium alloy by cathodic sputtering using the SPI Module Sputter Coater (SPI Supplies, West Chester, PA, USA). The SEM images were obtained using a secondary electron (SE) detector at 100× magnification and an acceleration voltage of 15 kV. The software used for the measurements was xT Microscope Control (version 4.1.10, Thermo Fisher Scientific, Hillsboro, OR, USA).

### 2.4. Antioxidant Activity

The study of the antioxidant activity of the by-products was performed by the ABTS colorimetric assay, based on the protocol reported by Zemljič et al. [24]. Briefly, 0.125 g of milled material was dispersed in 5 mL of the ABTS solution and allowed to react in the dark at room temperature for 30 min, before measuring the absorbance at 734 nm (UV-Vis spectrophotometer Lambda 35, PerkinElmer, Springfield, IL, USA). The results, expressed as a percentage of the ABTS radical cation (*ABTS^•+^*) inhibition, were calculated by the following equation, where *A_control_* is the absorbance of the *ABTS^•+^* solution (blank) and *A_sample_* is the absorbance of the by-products’ solutions:

$$\%\;{ABTS}^{•+}\;inhibition\;=\frac{A_{control}-A_{sample}}{A_{control}}\times 100\;$$

### 2.5. Statistical Analyses

GraphPad Prism 8 software (GraphPad Software Inc., San Diego, CA, USA) was used to perform statistical analyses. Parametric tests were applied, and to compare two or more groups, a one-way ANOVA was applied, followed by Tukey’s multiple comparison tests. Results are presented as overall mean ± SD, and statistical significance was defined as *p* < 0.05 for a 95% confidence interval.

## 3. Results and Discussion

### 3.1. Physicochemical Characterisation of the Plant-Based By-Products

#### 3.1.1. Chemical Composition and Thermal Stability of the Materials

**Figure 2:**
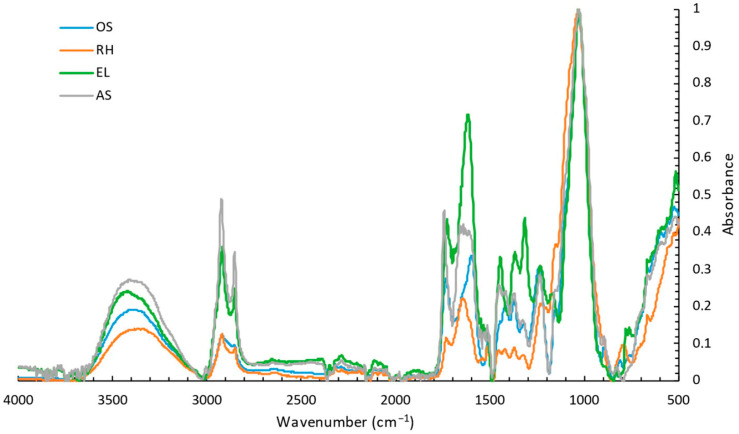
FTIR-ATR absorption spectra of the plant-based by-products, oregano stalks (OS), rice husk (RH), eucalyptus leaves (EL), and almond shells (AS).

The chemical characterisation of the plant-based by-products was performed by FTIR to analyse their molecular composition and structure (Figure 2). Considering the lignocellulosic nature of the materials in the study, the presence of spectral peaks or bands corresponding to cellulose, lignin, tannins, and oils was anticipated. The spectral intensity values were normalised with reference to the characteristic C-O, C-C, and C-C-O stretching vibration of cellulose at 1025–1035 cm^−1^. The prominent peaks in the range of 2916–2863 cm^−1^, characteristic of the asymmetric and symmetric CH_2_ stretching in oils, were identified at higher intensity in the EL and AS spectra. The cellulose content of a lignocellulosic material can be estimated using FTIR spectroscopy by analysing specific absorption bands associated with different molecular vibrations in cellulose. A broad band appears in the 3400–3300 cm^−1^ range due to the stretching of the OH groups, which play a key role in hydrogen bonding with cellulose. Weak bands were observed at 2940–2915 cm^−1^ corresponding to the antisymmetric stretching of the CH_2_ and at 2890–2880 cm^−1^ due to the symmetric stretching of the CH groups. Additionally, a weak band at 2870–2840 cm^−1^ due to the symmetric stretching of the CH_2_ was detected. Medium weak bands were detected at 1480–1430 cm^−1^ due to the deformation of the CH_2_ and at 1390–1350 cm^−1^ due to the deformation of the CH. A weak band was also observed at 1380–1300 cm^−1^ due to the deformation in-plane of the OH. The FTIR analyses revealed more medium-weak bands at 1330–1300 cm^−1^ due to the deformation of the CH_2_, at 1170–1130 cm^−1^, and at 1100 cm^−1^ due to the stretching of the C-O-C ether group. Moreover, a medium-strong band was observed at 1100–1000 cm^−1^ due to the stretching of the CO groups, and a weak band at 750–650 cm^−1^ due to the deformation out of plane of the OH groups. Additionally, the high-intensity peak at 1149 cm^−1^, which is common in all studied materials, is associated with the C-H stretching in the lignin structure. The presence of tannins is acknowledged by the presence of peaks in the range of 1400–1600 cm^−1^ of the spectra [25,26].

To estimate the chemical composition of the materials, the percentage of cellulose, lignin, tannins, and oils was calculated (Table 1) as the sum of the areas of the characteristic bands of each component divided by the total area of the spectrum, following the same strategy developed previously [26]. The results presented are in accordance with previous reports from the literature, which confirm the richness of the studied materials in cellulose and lignin [27,28,29]. Despite the similarity and high cellulose and lignin content in all the plant-based by-products, RH presents a higher cellulose content, while EL and AS present a higher percentage of oils. Interestingly, EL presents higher values of extractable tannins compared to the others. Tannins can play a significant role in a wide range of industries, namely textile, health, and pharmaceutical [30]. These compounds are currently used in leather tanning, and their integration into fireproofing and insulation foams is being explored to replace petroleum-based additives. The presence of extractable tannins in the biomaterials analysed suggests possible applications in foam production, taking into consideration their composition. They can be exploited for their medicinal and antioxidant potential, as inhibitors of harmful pro-oxidant enzymes and as antibacterial agents for hospital textiles, as well as in sports or domestic clothing to prevent odour formation [30,31].

**Figure 3:**
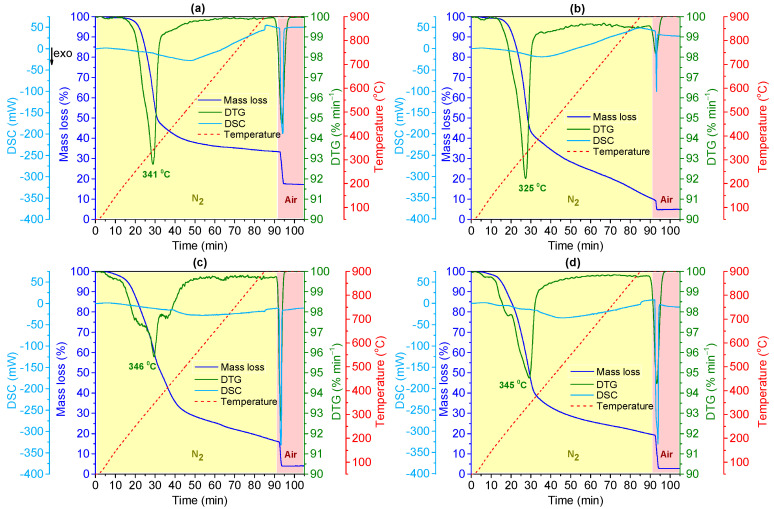
Thermograms coupled with DSC and DTG curves of plant-based by-products: (**a**) RH, (**b**) OS, (**c**) EL, and (**d**) AS. ‘↓exo’ indicates that exothermic events are identified at DSC values below 0 mW.

The thermal stability and behaviour were assessed by TGA-DSC. The resulting TGA-DSC profiles, along with the first derivative of the TG curves (DTG), are presented in Figure 3. Furthermore, the inert atmosphere (N_2_) is marked in the yellow-shaded region and the oxidative atmosphere (air) in the pink-shaded region. The peaks observed in the DTG curve correspond to the inflection points of the TG curves, indicating mass loss events. By calculating the minimum point of each DTG peak, it was possible to determine the temperature at which the mass loss occurred.

Along with TGA-DSC and DTG profiles, Table 2 includes the moisture content, volatile compounds, fixed carbon, and ash of plant-based materials after the drying process.

By analysing the TGA-DSC profiles, a mass reduction was observed between 250 and 380 °C, which is related to the rapid decomposition of cellulose, hemicellulose, and lignin—pyrolysis—resulting in the formation of volatile compounds such as water, carbon monoxide, carbon dioxide, and various organic compounds. This phase is characterised by the breakdown of the cellulose polymer into smaller molecules and gases [32]. The observed continuous mass loss corresponds to lignin degradation, which is characterised by lower reaction kinetics and occurs over a broad temperature range, producing char [33]. Then, changing the atmosphere to an oxidising one, the burning of all organic material can be seen at 900 °C, with DSC peaks defined at values < 0 mW, indicating exothermic reactions during this process. Concerning the moisture content, the TGA confirmed that the applied drying protocol (60 °C for 3 h) effectively reduced the moisture content of all plant-based materials to low levels. Since each plant-based material has differences in chemical composition, the amounts of formed volatile compounds, fixed carbon, and ash were also different. For instance, OS was found to have a higher percentage of volatile compounds (90.6 wt.%) and showed the lowest fraction of fixed carbon (4.84 wt.%), explaining the lower energy release (DSC peak intensity) during the exothermic burning process at 900 °C.

Considering that the residues characterised by TGA-DSC showed considerable values of inorganic matter, a subsequent ICP-OES analysis of all residues was performed.

The elemental analysis of the residues was performed to determine the quantity of C, H, N, S, and O within each of the materials. The results from the elemental analysis are presented in Table 3, which indicates the mass fractions of C, H, N, S, and O in the studied materials. Considering the results obtained from TGA, it is possible to confirm that the EA analyses were performed on a dry-free basis and permitted to convert the results into a dry ash-free basis, approximating the standard ISO 16948:2015 recommendations. By the analysis of the results, it is possible to conclude that the plant-based by-products have a similar chemical composition. Oxygen content was both directly measured using an OXY cube analyser and calculated by difference following ISO 16948:2015. The results generally showed good agreement, with minor deviations observed between the two approaches. However, in the case of RH, a larger discrepancy (~8 wt.%) was noted. This deviation may be attributed to matrix effects, particularly due to the high ash content observed for this material (17 wt.%). The presence of significant inorganic matter could contribute to an overestimation of the oxygen content measured via elemental analysis, potentially due to partial oxidation of inorganic compounds during the analysis process. This highlights the importance of presenting both values to assess the robustness of the elemental characterisation.

#### 3.1.2. Chlorine Content

The determination of chlorine content in the plant-based materials was accessed by IC analyses by measuring the presence of the anion chlorine in the previously digested samples. The obtained results confirmed that chlorine could be present at very low amounts since the concentrations of it are below the limit of detection for all the materials, as expected [34,35,36,37]. However, since the detection limit is 0.452 ppm—corresponding to 0.05 wt.% under the conditions established in this analysis—it exceeds the admissible limits for certification that permit the use of these materials for heating purposes, as the established limits range between 0.02 and 0.03 wt.% (dry mass). Further studies must be conducted to precisely quantify this element if the intended application involves heating, in order to address the previously mentioned limitations related to the presence of chlorine in biomass.

#### 3.1.3. Metals Assessment

The metal content of the dried plant-based by-products was evaluated using ICP-OES, and a total of 47 metal elements were analysed. However, only the elements detected in measurable concentrations are reported, including B, Ca, Fe, K, Mg, Na, P, and Zn, as shown in (Table 4), since the concentration of the other elements was below the limit of detection. Among these, Ca and K were the metals at higher concentrations, as expected, considering these metals are present in the typical chemical composition of plant biomass macronutrients [38,39,40,41].

#### 3.1.4. Wetting Behaviour and Hydrophobicity

**Figure 4:**
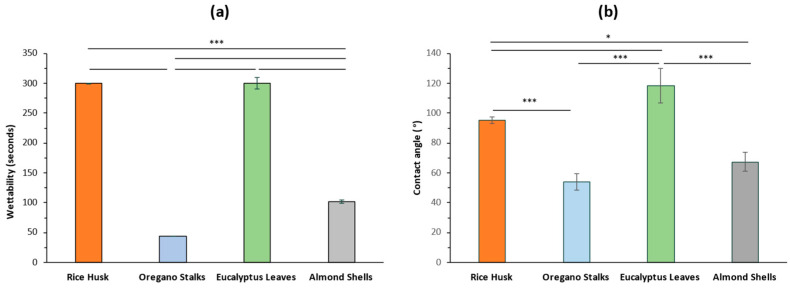
Time taken by the by-products to penetrate the water surface in wettability tests (**a**) and contact angle measurements of the residues (**b**). Results are presented as mean ± SD, and statistical differences are noted as * *p* < 0.05, *** *p* < 0.001.

The wettability and hydrophobicity of the studied residues were also assessed (Figure 4). Among the residues, EL and RH exhibited the lowest wettability, for which more than 300 s were necessary to penetrate the water surface, compared to the 44 and 102 s observed for OS (Figure 4a). Interestingly, the residues with lower wettability were those with higher oil and lignin content, which have been associated with a hydrophobic nature. Hydrophobicity was further evaluated through contact angle measurements, where EL presented a contact angle of 118°, indicating a highly hydrophobic profile, consistent with the wettability results. In contrast, the remaining materials showed lower hydrophobicity, with 54°, 95°, and 67° contact angles for OS, RH, and AS, respectively (Figure 4b). Hydrophobicity is a solid surface property that allows substances to repel water, and surfaces with a water contact angle greater than 90° are termed hydrophobic. In this study, EL and RH, with contact angles above 90°, are considered hydrophobic and can be incorporated into hydrophobic coatings. When combined with suitable binders, these materials have potential applications in industries such as textile coating.

#### 3.1.5. Morphological Analysis of the Plant-Based By-Products

**Figure 5:**
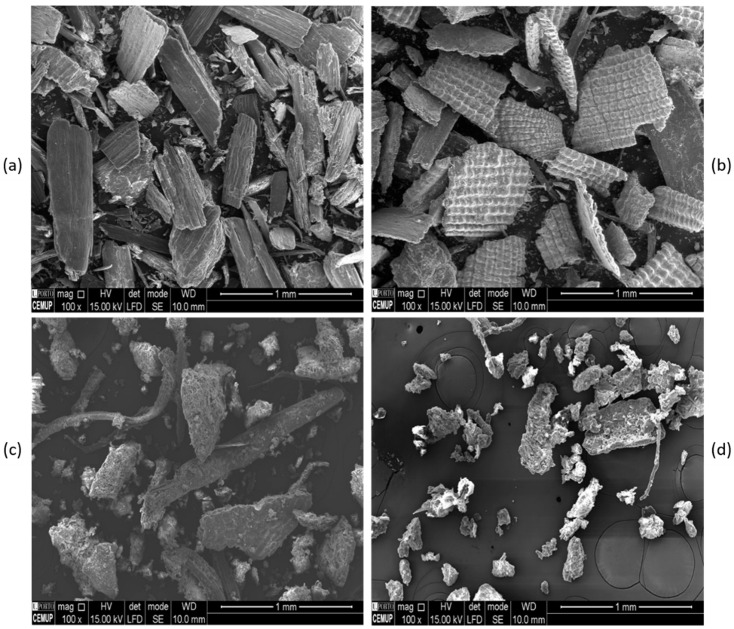
SEM images at 100× magnification of the residues (**a**) OS, (**b**) RH, (**c**) EL, and (**d**) AS.

The morphological analysis of the dried materials was performed by SEM at 100× magnification to visualise the different milled residues (Figure 5). The OS and AS particles exhibit fibrous characteristics with heterogeneous sizes. In contrast, EL shows a mixture of fibrous and more amorphous particles, while AS is composed of smaller particles with irregular shapes.

Analysis of the particle sizes was also conducted, and approximately 11 measurements of particle size were taken per residue analysed. The average particle size and standard deviation are presented in Table 5. A significant variability was observed, indicating a high polydispersity in particle size. The OS exhibited the largest average particle size (637.5 µm) along with the highest polydispersity, whereas the AS displayed the smallest average particle size (82.0 µm).

### 3.2. Antioxidant Activity and Assessment of Stability over Time

**Figure 6:**
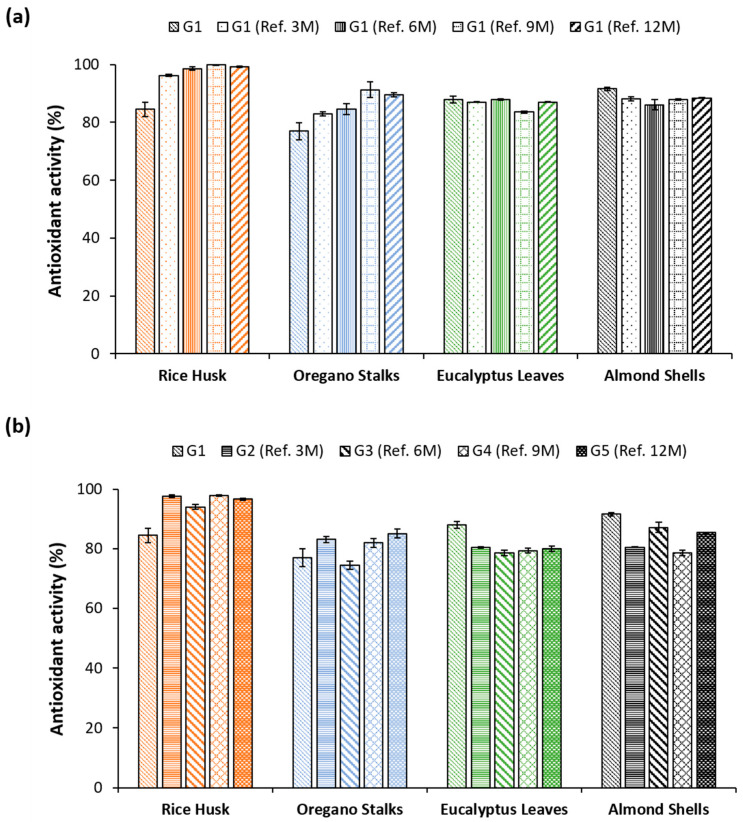
The antioxidant activity of plant-based by-products ground at day 0 (G1) and measured after 3 (G1 Ref. 3M), 6 (G1 Ref. 6M), 9 (G1 Ref. 9M), and 12 (G1 Ref. 12M) months (**a**). Antioxidant activity of the same residues milled only on assay day and stored for 3 (G2 Ref. 3M), 6 (G3 Ref. 6M), 9 (G4 Ref. 9M), and 12 (G5 Ref. 12M) months (**b**). Results are expressed in a percentage of the *ABTS*^•+^ inhibition and refer to the mean and standard deviation.

The ABTS method was used to evaluate the antioxidant activity of the residues under study. Their antioxidant activity was evaluated immediately upon reception (G1). Thereafter, the residues were stored as received (unprocessed) and also milled to 0.25 mm, and the antioxidant activity of both forms was monitored over the storage period, with measurements taken at 3, 6, 9, and 12 months (G2, G3, G4, and G5, in Figure 6). The unprocessed residues were milled to 0.25 mm only on the day of the antioxidant activity assay.

All the stored milled residues showcased antioxidant activity (>80%) that was maintained over the 12 months (Figure 6a). The RH presented the strongest activity, with an activity above 95%, maintained after 12 months of storage. Similar results were observed for the unprocessed stored residues, with an observed antioxidant activity above 75% (Figure 6b). The antioxidant activity was observed over time, showcasing that the studied residues can maintain their antioxidant properties for at least 12 months independently of the storage granulometry and the use of mechanical processing. These results are important in an industrial context, supporting the processing of the plant-based by-products to a smaller granulometry and yielding a smaller volume that facilitates the storage of the residues.

## 4. Conclusions

The physicochemical characterisation of the four plant-based by-products revealed that these materials present a cellulose- and lignin-based composition, both structural molecules with a significant range of applications across different industries, such as applications for biocomposites, packaging, and functional coatings. Additionally, the hydrophobic nature of the EL, together with its low wettability, suggests its potential for hydrophobic applications, ranging from construction materials (e.g., composites and insulation systems) to the textile industry (e.g., water-resistant fabrics). Moreover, all the residues presented antioxidant activity across a 12-month period that was not affected by the mechanical processing of the residues. In this way, the good thermal and antioxidant stability of the samples indicates their potential to be used in industrial applications, where the long-term storage of raw residues is crucial.

This study showcases the potential of these plant-based materials, which are currently considered waste and discarded by the industry. Their composition and properties of interest indicate that these materials can be reclassified as by-products, improving resource efficiency and promoting a circular economy.

The identification of new residues and the characterisation of their full composition and properties are critical to identifying and selecting new materials, potentiating a new economic value chain for these waste materials.

## Figures and Tables

**Table 1:** Percentage of cellulose, lignin, tannins, and oils in the studied organic materials.

Sample	Cellulose (%)	Lignin (%)	Extractable Tannins (%)	Oils (%)
OS	38	15	10	3
RH	52	14	7	3
EL	33	26	14	6
AS	34	18	11	8

**Table 2:** Humidity values, volatile compounds, fixed carbon, and ash obtained through TGA analysis.

Sample	Humidity (wt.%)	Volatiles (wt.%)	Fixed Carbon (wt.%)	Ash (wt.%)
RH	0.24	66.7	16.3	17.0
OS	0.40	90.6	4.84	4.53
EL	0.29	84.2	12.0	3.78
AS	0.45	81.0	16.7	2.28

**Table 3:** Elemental analysis of plant-based by-products in terms of C, H, N, S, and O on a dry ash-free basis, considering the ash content collected from TGA-DSC analyses.

Sample	C (wt.%)	H (wt.%)	N (wt.%)	S (wt.%)	O (wt.%)	O (wt.%) ^ISO^
RH	45.5	6.11	0.72	0.01	47.9	39.6
OS	43.8	5.81	0.50	0.01	46.7	47.6
EL	52.6	6.70	1.12	0.00	38.3	38.1
AS	48.0	6.43	1.08	0.01	42.4	43.4

^ISO^: Oxygen content calculated following the ISO 16948:2015 standard requirements.

**Table 4:** ICP-OES analyses of RH, OS, EL, and AS residues.

Metal (wt.%)	RH	OS	EL	AS
B	0.40	0.00	0.00	0.51
Ca	6.36	0.91	1.07	4.16
Fe	0.46	0.00	0.02	0.00
K	2.87	1.49	0.49	1.97
Mg	0.98	0.05	0.22	0.06
Na	0.39	0.04	0.19	0.04
P	0.57	0.20	0.42	0.57
Zn	0.67	0.00	0.00	0.04

**Table 5:** Average size of the plant-based by-products, determined by SEM analyses.

Sample	Average Particle Size (µm)	Standard Deviation (µm)
RH	420.5	263.9
OS	637.5	550.0
EL	244.2	230.5
AS	82.0	73.2

## Data Availability

The data that support the findings of this study are available from the authors upon reasonable request due to institutional data use policies.
